# Attitudes of mental health clinicians toward perceived inaccuracy of a schizophrenia diagnosis in routine clinical practice

**DOI:** 10.1186/s12888-018-1897-2

**Published:** 2018-09-27

**Authors:** Dana Tzur Bitan, Ariella Grossman Giron, Gady Alon, Shlomo Mendlovic, Yuval Bloch, Aviv Segev

**Affiliations:** 1 Department of Behavioral Sciences, Ariel University, Ramat Hagolan 65th st, 4070000 Ariel, Israel; 2 Shalvata Mental Health Center, 13th Alyat Hanoar st, POB 94, 45100 Hod Hasharon, Israel; 3 Sackler School of Medicine, Tel Aviv University, POB 39040, 69978 Tel Aviv, Israel

**Keywords:** Schizophrenia, DSM, ICD, Misdiagnosis, Rehabilitation, Negative symptoms

## Abstract

**Background:**

Mental health clinicians have previously been reported to express reservations regarding the utility and accuracy of the psychiatric classification systems. In this study we aimed to examine clinicians’ experiences with instances of perceived inaccuracy of a schizophrenia diagnosis.

**Methods:**

Mental health clinicians *(N =* 175) participated in an online survey assessing prevalence and perceived reasons for inaccuracies of a schizophrenia diagnosis. Respondents included psychiatric ward directors (13.1%), senior psychiatrists and psychologists (40.5%), and psychiatry and clinical psychology residents (36%).

**Results:**

Fifty-three percent of respondents reported encountering instances where a schizophrenia diagnosis was assigned even though clinical presentation did not match diagnostic criteria. Seventy-three percent of senior psychiatrists in a position to determine a diagnosis declared assigning schizophrenia even when controversial among clinical staff, and 15% of them declared doing so frequently. The likelihood of frequently assigning a schizophrenia diagnosis even when clearly controversial was predicted by the perception that an inaccurate diagnosis is assigned due to the presence of negative symptoms (OR 2.20, 95% CI 1.04–4.66, *p* = 0.039) and due to patient-related factors, such as the need to facilitate rehabilitation (OR 1.77, 95% CI 1.07–2.90, *p* = 0.024).

**Conclusions:**

Although a schizophrenia diagnosis is considered relatively stable and clear, our study indicates that, in clinical practice, the assignment of this diagnosis is frequently controversial. These controversies are associated with the perception that an inaccurate diagnosis is assigned due to diagnostic considerations, or due to the possibility that patients might benefit from such a diagnosis. Implications and limitations for psychiatric practice and discourse are discussed.

## Background

Surveys examining mental health clinicians’ attitudes towards the classification systems indicate that mental health professionals have different views of their utility and accuracy. For example, a global survey found that psychiatrists rated the ICD diagnostic criteria to be fairly accurate and easy to use [1]. On the other hand, a World Health Organization survey found that psychologists rated several diagnostic criteria as low on goodness of fit as compared to ease of use, suggesting that they view some problems with the descriptive validity of the classification systems [2]. It was also reported that although more than 90% of psychologists use the DSM for classification purposes, most of them express serious concerns about various aspects of the diagnostic system [3].

The study of mental health clinicians’ attitudes towards the accuracy of psychiatric classification is even more meaningful in the case of a schizophrenia diagnosis. Studies indicate that the label of schizophrenia in and of itself not only affects mental health and primary care providers’ expectations (such as patients’ adherence and ability to manage their illness), but also their actual treatment decisions [4]. Additional byproducts of schizophrenia labeling include self and social stigma which, regardless of illness severity, can facilitate a severe course and prognosis, and imperil chances of recovery [5–11]. Taking these implications under consideration, it is reasonable to assume that clinicians’ security with assigning such a diagnosis should be relatively high.

Surprisingly, not many studies have assessed clinicians’ perceptions about the accuracy and inaccuracy of a schizophrenia diagnosis as utilized in routine clinical settings. A relatively modest indication of clinicians’ attitudes towards instances of schizophrenia misdiagnosis can be found in a study [12] reporting that almost 50% of surveyed psychiatrists believed that schizophrenia tends to be frequently misdiagnosed among black people. Indeed, studies addressing schizophrenia diagnosis inaccuracy have mainly concentrated on social and diagnostic factors associated with over, under, or misdiagnosis of schizophrenia, such as difficulties in differentiating schizophrenia from either axis 1 or 2 diagnoses [13–18], or misdiagnosis due to immigration or racial influences [19–22]. To the best of our knowledge, no study has systematically explored how frequently mental health clinicians encounter, or take part in, labeling patients with the diagnosis of schizophrenia, even when such a label is either controversial or subjectively perceived to be inaccurate.

In this study, we aimed to bridge this gap by examining mental health clinicians’ experiences with the assignment of a schizophrenia diagnosis. Specifically, we focused on three research questions: (a) How often do mental health clinicians participate in assigning a schizophrenia diagnosis even when clinical presentation does not match diagnostic criteria? (b) How often do mental health clinicians perceive a schizophrenia diagnosis to be inaccurate even when clinical presentation matches diagnostic criteria? (c) How often do senior psychiatrists, authorized to assign a schizophrenia diagnosis, assign such a diagnosis even if controversial among clinical staff? Additionally, we aimed to explore whether the assignment of a controversial schizophrenia diagnosis is associated with specific attitudes regarding the underlying reasons for the occurrence of such instances.

## Methods

### Participants

Participants were psychiatrists, psychologists, and social workers who were on the mailing lists of any of three mental health centers in Israel: Shalvata Mental Health Center, Lev Hasharon Mental Health Center, and Geha Mental Health Center. These three centers are characterized by a diagnostic process which frequently, but not always, involves an interdisciplinary staff meeting during patient interviewing (intake) in order to establish diagnosis. Therefore, clinicians at these centers were viewed as relevant participants for the current study. During these meetings, staff discussion is usually recorded in patients’ clinical files. Information regarding the patient’s diagnosis is usually shared sensitively and cautiously after the diagnosis has been assigned, and in most cases the patient does not participate in the discussion related to his/her diagnosis. The survey was also circulated in the department of psychiatry at the school of medicine of the affiliated university, as well as two classes of the psychotherapy school of the affiliated university.

### Procedure

The study was reviewed and authorized by the Institutional Review Board of the Shalvata Mental Health Center in Israel. Participants were recruited through an e-mail containing an embedded link to a Qualtrics electronic survey. The participants were informed about the subject of the study, the estimated time for its completion, and the safeguarding of anonymity, and were also informed that answering the questionnaire signified their agreement to participate in the study. Demographic and clinical characteristics were collected, and were followed by items inquiring about experiences of perceived inaccuracy of schizophrenia diagnoses in clinical settings. A 20-item questionnaire, specifically designed to assess perceptions regarding the underlying reasons for inaccuracy in schizophrenia diagnosis, followed. Participants’ responses were coded using designated research codes, and their identities were completely anonymized.

### Construction of the questionnaire assessing reasons for inaccuracy in schizophrenia diagnosis

The uniquely designed questionnaire assessing reasons for inaccuracy in schizophrenia diagnosis comprised three sections (see Appendix A). The first part obtained demographic data (age, years of experience, position, exposure to patients with schizophrenia). In the second part, participants were requested to report the frequency with which they experienced instances of inaccurate diagnosis of schizophrenia. As schizophrenia is viewed to be a relatively clear diagnosis [23], we assumed that even frequent cases of inaccurate diagnosis would rarely rise above the 10% mark. Therefore, the questions and associated categories were constructed as follows: (a) How often did you participate in assigning patients a diagnosis of schizophrenia even though clinical presentation did not meet the DSM criteria (as part of a staff meeting)? Answers were *never*, *rarely* (less than 1% of cases), *occasionally* (1–5%), *frequently* (5–10%), and *very frequently* (more than 10%); (b) How often did you disagree with the assigned schizophrenia diagnosis despite the matched DSM criteria? (c) If you are a senior psychiatrist, and authorized to assign and document a diagnosis, how often have you given a diagnosis of schizophrenia even though other staff members opposed such a diagnosis? The rationale for using the DSM (rather than the ICD) as common ground for diagnosis assignment was guided by the need to use a diagnostic system relatively familiar to all clinical sectors, and to serve as an archetype for any diagnostic system that is set to provide a formal diagnosis. The third section included 20 statements referring to possible reasons for inaccurate diagnosis. These statements were generated based on previous literature associating the inaccuracy of a schizophrenia diagnosis with specific diagnoses or agendas [24–27]. For example, as borderline patients are often hospitalized for prolonged periods of time due to psychotic episodes [24], which might in turn serve as a basis for inaccurate schizophrenia diagnoses, statements referring to such possible associations were constructed. For example: “A significant percentage of the patients were characterized by a tendency towards self-injury” and “A significant percentage of these patients were characterized by frequent or prolonged hospitalizations.” Answers were given on a 5-point Likert-type scale (ranging from *strongly agree* to *strongly disagree*), reflecting participants’ opinions regarding the reasons they felt contributed to an inaccurate diagnosis of schizophrenia. The statements pertain to symptom-based characteristics (deficit, affect, emotional dysregulation, use of drugs, etc.); management-based characteristics (frequent admissions, drug-resistance, rehabilitation, etc.); and staff-based characteristics (experience, burnout, etc.).

## Results

Demographic and clinical characteristics of the mental health clinicians participating in the survey are presented in Table 1. A total of 175 clinicians responded to the survey. Thirteen percent of the participants were psychiatric ward directors, 71 (about 40%) were senior psychiatrists and psychologists, 32 (18%) were senior psychiatry residents (already authorized to determine psychiatric diagnoses and treat independently in a private clinic setting, though not in a hospital). The mean years of experience in the clinical field was 11.05 (*SD* = 9.80). About half of the participants were employed (either part or full-time) in psychiatric hospital wards (47.1%), and 52.9% were employed (either part of full-time) in public outpatient clinics. The vast majority of participants reported working with patients diagnosed with schizophrenia either very frequently (56%) or frequently (17.7%). A large proportion of the participants reported that they engaged, with varying frequency, in writing legal psychiatric opinions, and 35% of respondents declared that they engaged in writing legal psychiatric opinions as part of their daily work.

**Table 1:** Characteristics of clinicians responding to the survey

Main demographic and clinical characteristics		*Mean (SD)*	Frequency (*%*)
Age		41.59 (8.99)	
Experience (in years)		11.05(9.80)	
Gender	Male		69 (39.4%)
	Female		106(60.6%)
Position	Psychiatric Ward Director		23 (13.1%)
	Senior Psychiatrist		37 (21.1%)
	Senior Psychiatry Resident		32 (18.3%)
	Junior Psychiatry Resident		17 (9.7%)
	Senior Clinical Psychologist		34 (19.4%)
	Clinical Psychology Intern		14 (8%)
	Social Worker		18 (10.3%)
Clinical work place	Hospital ward		82 (47.1%)
	Public outpatient clinic		92 (52.9%)
	Private clinic		68 (39.0%)
	Other		13 (7.5%)
Writing legal Psychiatric opinion	Frequently		62(35.4%)
	Rarely		42 (24%)
	Never		70 (40%)
Working with Schizophrenia Patients	Very Frequently		98 (56%)
	Frequently		31 (17.7%)
	Not often		18 (10.3%)
	Rarely		28 (16%)

Responses of clinicians from different clinical positions to questions concerning the perceived accuracy of a schizophrenia diagnosis in daily practice are presented in Table 2.

**Table 2:** Frequency distribution of perceived accuracy of a schizophrenia diagnosis

	Psychiatrists *N* = 109 N (%)	Psychologists *N* = 48 N (%)	Others *N* = 18 N (%)	Total *N* = 175 N (%)	Chi Square
Gave SCZ diagnosis when clinical presentation did not match DSM?					8.56
Never	42 (38.5%)	30 (62.5%)	9 (50%)	81 (46.3%)	
Rarely	52 (47.7%)	15 (31.3%)	8 (44.4%)	75 (42.9%)	
Frequently	15 (13.8%)	3 (6.3%)	1 (5.6%)	19 (10.9%)	
Disagreed about SCZ diagnosis despite matched DSM criteria?					11.95*
Never	30 (27.8%)	24 (50%)	11 (61.1%)	65 (37.1%)	
Rarely	62 (57.4%)	19 (39.6%)	6 (33.3%)	87 (49.7%)	
Frequently	16 (14.8%)	5 (10.4%)	1 (5.6%)	22 (12.6%)	
Gave SCZ diagnosis when controversial? (authorized psychiatrists only)					n/a
Never	16 (26.7%)			16 (26.7%)	
Rarely	35 (58.3%)			35 (58.3%)	
Frequently	9 (15.0%)			9 (15.0%)	

SCZ = Schizophrenia; DSM = Diagnostic and Statistical Manual of Mental Disorders. *p < .05

Most respondents reported giving a diagnosis of schizophrenia even when the clinical presentation of the patient did not match DSM criteria; out of those, 42.9% reported encountering such situations rarely and 10.9% reported encountering such situations frequently. There was no significant difference between the different clinical positions. Most respondents also reported that they experienced subjective disagreement with the diagnosis even when the clinical presentation met the diagnostic criteria, either rarely (49.7%) or frequently (12.6%), with a statistically significant difference among the clinical positions. A Bonferroni correction post-hoc analysis (at alpha level of 0.005) indicated a lower frequency of psychiatrists reporting that they never had a disagreement regarding a schizophrenia diagnosis (adjusted standardized residual *Z* = − 3.3, *p* < 0.005). Finally, among the senior psychiatrists (34.2% of the entire sample), 58.3% declared assigning a schizophrenia diagnosis even if controversial among clinical staff, and 15% declared encountering such situations frequently.

A principal component analysis (PCA) was next performed on the entire sample of clinicians in order to cluster the various reasons for the perceived inaccurate diagnosis. Tests for sampling adequacy included the Kaiser-Meyer-Olkin, which produced a value of 0.75, and the Bartlett test of sphericity, which was statistically significant (*p* < .001). These values are well within range [28–30] and point to the suitability of the data for factor analysis. As the factors were assumed to be interrelated, oblique rotation was used. The PCA resulted in a six-factor solution explaining 64.73% of the variance. Four factors were retained from this analysis based on the number of highly (i.e., .40 and higher) and uniquely loaded items per factor and interpretability, resulting in one item which was excluded from analysis. These factors were found to have the following thematic organization: (A) Staff-related considerations. These included items reflecting different emotional aspects of the staff’s clinical routine, such as attempting to resolve daily clinical decisions which are hard to make due to unclear diagnosis, occupational fatigue, alleviation of staff’s feelings of helplessness, and conceptions of senior psychiatrists. (B) Patient-related considerations. These included items reflecting patients’ therapeutic and emotional processes, such as helping patients come to terms with their severe mental states, helping patients obtain their social rights, facilitating rehabilitation programs; (C) Borderline in differential diagnosis. These included items reflecting signs and symptoms that suggest that the diagnosis should be a severe form of borderline personality disorder, such as multiple hospitalizations, tendency to self-harm, difficulties in emotional regulation, recurrent episodes of affective episodes, and treatment-resistant patients; and (D) Negative symptoms schizophrenia, which included the following items: the existence of severe and prolonged functional impairment, the existence of a significant impairment in affect, and excessive hospitalizations.

On the basis of these four factors, scale scores were then calculated by averaging the items for each scale. Means, *SD*s, Cronbach alpha coefficients, and Pearson correlations for the resulting four scores are presented in Table 3.

**Table 3:** Means, standard deviations, alpha reliability coefficients, and Pearson correlations for identified four factors

	Mean	Standard Deviation	Alpha	A	B	C
A. Staff-related factors	2.95	0.73	0.80			
B. Patient-related factors	2.61	0.72	0.74	.21^*^		
C. Borderline in differential diagnosis	2.96	0.78	0.80	.45^**^	.26^**^	
D. Presence of negative symptoms	3.35	0.67	0.56	.35^**^	.25^**^	.35^**^

* *p* < .05 ** *p* < .01

As can be seen, most scales had means slightly below the middle point of the five-point scale, and the *SD*s all indicate reasonable variance. The Cronbach alpha reliability coefficients ranged from 0.56 to 0.80. Correlations between the scales were all statistically significant and positive and ranged from 0.21 to 0.45.

Finally, we aimed to explore the association between the perceived reasons for inaccuracy in schizophrenia diagnosis and the assignment of a schizophrenia diagnosis by senior psychiatrists even when controversial and opposed by the clinical staff. Binary logistic regression was performed with the four factors serving as predicting variables, while the probability of frequently giving a schizophrenia diagnosis even when controversial or opposed by the clinical staff served as the dependent variable. Age and years of experience were entered as covariates. The analyses yielded a significant model, *χ*^2^ (6) = 18.82, *p* < .01, explaining between 31.9% (Cox & Snell R-squared) and 57% (Nagelkerke R-squared) of the variance of frequent assignment of a controversial diagnosis. Results of the logistic regression analysis are presented in Table 4. Results indicate that perceiving inaccuracy in a schizophrenia diagnosis as emerging from the presence of negative symptoms, or from patient-related factors, significantly predicted the probability of making a schizophrenia diagnosis even when controversial.

**Table 4:** Logistic regression for the prediction of frequently giving a schizophrenia diagnosis even when controversial

	Odds Ratio	Confidence Interval	*p value*
Staff-related factors	1.08	0.80–1.46	> 0.1
Patient-related factors	1.77	1.07–2.90	0.039
Borderline in differential diagnosis	0.95	0.59–1.52	> 0.1
Presence of negative symptoms	2.20	1.04–4.66	0.024

## Discussion

This is the first study attempting to depict clinicians’ experiences with situations in which a diagnosis of schizophrenia is perceived as inaccurate. Over 50% of the mental health clinicians assessed in our study reported assigning a schizophrenia diagnosis even when DSM criteria were not met, and about 10% of them reported such events to be frequent. In addition, 49.7% reported disagreements regarding diagnosis even when patients’ clinical presentation matched DSM criteria, with 12.6% estimating that these disagreements occur frequently. Finally, the majority of senior psychiatrists authorized to assign a schizophrenia diagnosis reported assigning such a diagnosis even when controversial among the clinical staff, either rarely or frequently. It is important to note that the participants in our study were mainly experienced mental health clinicians, many of them in teaching or clinical mentoring positions while working in team-based settings. Moreover, a large proportion of the study’s sample engaged in writing legal psychiatric opinions, where the assignment of a schizophrenia diagnosis can have a substantial impact.

Although the diagnosis of schizophrenia is considered stable and clear among expert clinicians [23, 31, 32], the current study suggests that among most mental health workers, the determination of such a diagnosis is not as clear as expected. Unlike general medicine, where laboratory tests or imaging procedures can corroborate or refute clinical diagnoses, psychiatric medicine still has no objective measures for diagnosis affirmation. As a result, the diagnostic system is forced to rely on phenomenological measures, which are known to be sensitive to subjective interpretation. This perspective has been previously addressed in the past, where scholars criticized the reliability-over-validity approach of the diagnostic system and the resulting “thinning out” of clinical psychiatry [33–37]. Our findings suggest that even though the revolution of the DSM-III was meant to minimize variability [33], the use of the classification system in team-based clinical settings, where interdisciplinary staff takes part in establishing a diagnosis, allows for more frequent occasions during which a diagnosis is either controversial or perceived as inaccurate. The issue of diagnostic validity can also be reflected by the conceptual differentiation between validity and utility, where the categorical classification of mental disorders as a whole is argued to suffer from validity issues, yet remains the most frequent form of diagnosis due to its utility in predicting course, prognosis, and outcome of a specific disorder [38, 39].

An additional interesting finding that emerged from the current study is that most of the psychiatrists reported disagreeing about schizophrenia diagnosis despite matched DSM criteria, either rarely or frequently. One possible explanation is that psychiatrists have an ambivalent attitude towards the standardization of routine clinical practice, forced on them by the adoption of the DSM. Drawing from interviews with psychiatrists, it was previously suggested [40] that a trend of “sociological ambivalence” exists among psychiatrists, where professionals in this field develop “workarounds,” in the form of alternative diagnostic typologies or diagnosis negotiations, in order to develop a sense of autonomy. Therefore, it is possible that the perception of inaccuracy stems from the ongoing negotiation between the need to develop a sense of autonomy in the provision of clinical diagnoses and the adherence to diagnostic standardization.

In order to assess the underlying attitudes toward inaccuracy in schizophrenia diagnosis, a measure of attitudes towards the origins of schizophrenia diagnosis inaccuracy was constructed. The results of the exploratory factor analysis indicated a four-dimension structure, which included (a) Staff-related factors: the perception that an inaccurate diagnosis is being assigned to aid the clinical staff by allowing them to strategize treatment and alleviate feelings of frustration and burnout; (b) Patient-related factors: the perception that an inaccurate diagnosis is often assigned due to the benefits that can accrue to the patient, ranging from the technical (welfare services, rehabilitation programs, etc.) to the psychological (acceptance of illness, the need for medication or supported housing, etc.); (c) Borderline in differential diagnosis: the perception that an inaccurate diagnosis of schizophrenia results from a severe form of borderline personality disorder, as expressed by impaired emotional regulation, self-harm tendencies, but also treatment-resistance and frequent or prolonged hospitalization, and (d) Presence of negative symptoms: the perception that an inaccurate diagnosis of schizophrenia results from the presence of negative symptoms, a diagnostic criterion of schizophrenia that is considered predominant in the former diagnosis of “schizophrenia simplex,” which no longer exists in the DSM but is acknowledged in the ICD-10 as simple-type schizophrenia.

When we explored the association between these emerged four factors and the probability of frequently assigning a schizophrenia diagnosis even when controversial, we found such an association in two main clusters: the first is the perception that an inaccurate diagnosis stems from negative-predominant schizophrenia, whereas the second refers to patient-related factors (clusters D and B, respectively). Senior psychiatrists who reported frequently assigning a schizophrenia diagnosis, even when it was controversial to do so, tended to view these instances as resulting either from the presence of negative symptoms or for the benefit of the patients. These senior psychiatrists are experienced clinicians with much familiarity and experience with previous diagnostic systems. Negative symptoms schizophrenia, a type which no longer exists in the DSM but is acknowledged in the ICD-10 as simple-type schizophrenia, is most probably very familiar to senior psychiatrists. Our findings may therefore indicate a certain lag in the implementation of the current version of the DSM-5, which adopted a stricter stance towards a schizophrenia diagnosis by the requirement of clear positive symptoms [41].

The second association found in our study is that senior psychiatrists who report frequently assigning a schizophrenia diagnosis even when controversial view the inaccurate diagnosis as a result of a process aimed at benefiting the patients. The concept of deliberate misdiagnosis, where a certain diagnosis is given even if not accurate in order to gain benefits, has been an issue for conceptual, clinical, and ethical debate. Namely, it has been suggested that the diagnostic labeling process is constantly shaped by cultural and policy environments, in which mental health services are provided under specific diagnostic criteria [42]. Studies indicate that social workers and physicians frequently face the dilemma of altering diagnoses due to procedural aspects of clinical care, such as the attainment of reimbursement [43–45]. Although warranting further research, our findings may suggest that this phenomenon occurs not only in the diagnosis of major depression [45] but also in the diagnosis of schizophrenia, as the probability of frequently assigning a schizophrenia diagnosis was associated with the view that an inaccurate diagnosis can result from such a process. Future studies are also needed to explore whether the reasons for inaccurate diagnosis stem from clinicians’ views and interpretations or, alternatively, derive from talking to service users. As the level of cooperation with service users during diagnosis assignment was not inquired about in the survey, an interesting line of research would be to explore how patients’ views might affect the perceived accuracy of the diagnostic process, as well as their own views on the assigned diagnosis.

It should be noted that the association between negative symptoms and patient-related factors and frequent assignment of controversial diagnoses does not necessarily imply that clinical staff endorsing such attitudes might themselves assign controversial diagnoses on the basis of such views. Instead, our findings represent perceived attitudes that can potentially reflect the clinicians’ views of the social and professional discourse towards the assignment of a schizophrenia diagnosis. The team-based approach to providing psychiatric diagnoses can be viewed as a natural ground for controversies, and can even be aimed at facilitating them. A process where a senior clinician makes a diagnosis, other members of the team question it, and a consensus then emerges, might be a valuable process, even in light of staff members’ reservations. Therefore, a critical line of future research that derives from our findings would be the study of the effects of such controversies on patients’ quality of care.

Several limitations should be noted. The cross-sectional design of our study does not allow for causality inferences. Future studies should assess, preferably by employing a longitudinal design, whether different views and perceptions of diagnosis inaccuracy can result in greater tolerance towards inaccurate diagnoses. As the factor structure of the scale assessing reasons for the inaccuracy of a schizophrenia diagnosis was derived from a sample of mental health clinicians, these factors can only be considered to apply to this population, and additional studies are needed to assess whether this factor structure applies across specific professional sectors. Additionally, although the study was performed in several clinical settings and across a variety of mental health professions, the sample size is considered modest. Cultural characteristics, whether social or professional, might have had an impact on study findings and may therefore affect the generalizability of our results. For example, professionals working primarily in mental health centers employing a team-based diagnostic process, such as the professionals who participated in this study, might encounter controversies more often than professionals who work in more independent settings. Additionally, the cultural and social atmosphere in Israel, and primarily the trend towards facilitating community integration of people with severe mental illness [46], might affect the level of sensitivity towards assigning a schizophrenia diagnosis, a factor which could vary across countries. Different mental health systems across the world vary in terms of professional staff, as well as of reliance on different classification systems (such as the ICD in most European countries), and can therefore present different patterns of views towards the level of accuracy of a given schizophrenia diagnosis. Further studies are needed in order to examine professionals’ views of diagnostic inaccuracy of schizophrenia in different cultural and social environments, as well as towards the different classification systems. Although the use of a survey in the current study allowed for intensive data collection and exploration of the main research questions, it should be noted that this approach is limited so as to primarily represent the explicit attitudes of participants, and therefore inferences cannot be made regarding associated potential behaviors or implicit views.

Finally, the use of volunteer sampling might have created a bias towards compliant mental health professionals; therefore, it is possible that clinicians who chose not to participate hold different views and attitudes.

## Conclusions

Despite the clear diagnostic criteria and reported stability of the schizophrenia diagnosis, our study indicates that mental health professionals’ experience of inaccurate labeling occurs more frequently than expected. The probability of assigning a schizophrenia diagnosis, even when controversial among staff, is associated with a perception of inaccuracy as resulting from either diagnostic considerations (the presence of negative symptoms), or for the benefit of the patient. The indication of negative symptoms and patient-centered factors as potential rationales for diagnostic inaccuracy might point to the need for a separate function-based diagnosis. These results may suggest a lag in implementing the new strict criteria of the DSM-5 to the assignment of such a diagnosis (primarily the exclusion of schizophrenia simplex), but may also reflect a mild ambivalence towards the classification system and a need for clinical autonomy. The use of a more general diagnostic label, such as “severe mental disorder involving a decline in adaptive functions,” might improve accuracy and agreement between professionals and help the clinical and research fields to improve both reliability and validity of psychiatric diagnoses, as well as improve public perception of psychiatry. Future studies are needed in order to explore these competing explanations. Finally, the association between assignment of an inaccurate diagnosis and the concept of doing so for the benefit of the patient might point to social, cultural, and policy effects on the process of diagnosis assignment. These effects should be further explored, as the current phenomenological structure of the classification system obligates professionals to rely on clinical evaluation, which is often subject to these effects. As a schizophrenia misdiagnosis can have severe clinical, social, occupational, and economic consequences, investigations into the routes leading to inaccuracy in diagnosis are highly essential. Such investigations can eventually allow for a more knowledgeable diagnostic process and reduce the prevalence of misdiagnosis in clinical settings.

## Appendix

**Table 5:** The questionnaire used in this study (translated to English)

Dimension	Item #	Description	Loading
Staff Related Factors	Q6	It is more likely that a misdiagnosis of Schizophrenia will be made by veteran caregivers	
	Q9	The diagnosis of Schizophrenia alleviated the clinical staff, in cases in which the treatment strategy was vague	
	Q16	The diagnosis of Schizophrenia might or has led to deterioration or impasse in these patients’ condition	
	Q17	I believe that feelings of helplessness or frustration in the clinical staff are a prominent factor in the misdiagnosis of Schizophrenia	
	Q18	I believe that the misdiagnosis of Schizophrenia is related, among other things, to feelings of burnout	
	Q19	Eventually, assigning the diagnosis of Schizophrenia alleviated the clinical staff	
Patient Related Factors	Q7	The diagnosis of Schizophrenia assisted the patient by facilitating various technical aspects (social financial aid, rehabilitation services, and medicinal re-imbursements)	
	Q8	The diagnosis of Schizophrenia assisted the patient for various emotional reasons (acceptance of his referral to rehabilitation services such as assisted living, supported employment, etc.)	
	Q15	Eventually, the diagnosis of Schizophrenia might or has contributed to the rehabilitation of patients in these cases	
	Q20	Eventually, assigning the diagnosis of Schizophrenia alleviated the patients	
Borderline-Like Patient	Q5	The patients in question were treatment resistant	
	Q10	A significant part of these patients were characterized by frequent or prolonged hospitalizations	
	Q11	A significant part of the patients were characterized by a tendency for self-injury	
	Q12	A significant part of these patients were characterized by major impairment in their capacity for emotional regulations	
	Q13	A significant part of the patients were characterized by repeating major affective episodes l	
Schizophrenia Simplex Type	Q1	The patients’ symptomatology did not sufficiently meet the DSM criteria of Schizophrenia	
	Q2	The Schizophrenia diagnosis was given due to severe and continuous impairment in functioning	
	Q3	The Schizophrenia diagnosis was given due to severe impairment in affect	
	Q4	A significant part of the patients were unnecessarily or redundantly hospitalized	

## Abbreviations

DSM: Diagnostic and Statistical manual for Mental Disorders
ICD: International Classification of Diseases
IRB: Institutional Review Board
PCA: Principal Component Analysis
SD: Standard Deviation
